# Research Advances of Neuroregulatory Effects of Dietary Polyphenols on Obesity Complications

**DOI:** 10.3390/nu18071075

**Published:** 2026-03-27

**Authors:** Tingting Han, Limeng Wei, Wei Gu, Sen Zheng, Yiqun Du, Huifang Ge, Daxiang Li, Zhongwen Xie

**Affiliations:** 1 National Key Laboratory of Tea Plant Germplasm Innovation and Resource Utilization, School of Tea Sciences, Anhui Agricultural University, Hefei 230036, China; tings_han@163.com (T.H.); zhengsen95@163.com (S.Z.); duyiqun@ahau.edu.cn (Y.D.); ghfjulia@ahau.edu.cn (H.G.); dxli@ahau.edu.cn (D.L.); 2 Department of Oncology, The First Affiliated Hospital of Anhui Medical University, Hefei 230022, China; wlm5511001@163.com; 3 Laboratory Animal Center, Anhui Medical University, Hefei 230032, China; guwei_public@163.com

**Keywords:** obesity complications, brain, gut–brain axis, dietary polyphenols

## Abstract

Background: Obesity is a chronic metabolic disease that has emerged as a major global public health concern. Obesity complications refer to a range of metabolic, neurological and behavioral disorders. Complex interaction mechanisms exist between obesity and the brain, including neuroendocrine regulation, center inflammatory responses, the gut–brain axis, and obesity-related cognitive impairment. Polyphenols are naturally occurring bioactive compounds widely found in plants. Recent research indicates that polyphenols may modulate the brain through multiple pathways, thereby ameliorating obesity complications. However, no data set available to summarize neuroregulatory effects of dietary polyphenols on obesity complication. Methods: The latest data available were collected to review research progress focusing on neuroregulatory roles of polyphenols on obesity complication. Results: This review summarizes the interaction between obesity and the brain and further explores the effects of polyphenols on obesity-related neurological disorders, with particular emphasis on their roles in appetite regulation, central neuroinflammation, brain leptin and insulin resistance, gut–brain axis modulation, and cognitive improvement. Finally, future perspectives are discussed. Conclusions: This paper may provide a new theoretical support and research direction for the potential of polyphenols against obesity-related neurological complications.

## 1. Introduction

Obesity has emerged as a major global health concern in recent decades. It is a major contributing factor to various chronic diseases, including cardiovascular disease, type 2 diabetes, and cancer, posing a serious threat to public health. According to the latest data from the World Health Organization, over 1 billion people worldwide were living with obesity in 2022, with about 890 million adults and 160 million children and adolescents between the ages of 5 and 19 affected [1]. This trend presents a substantial challenge to healthcare systems worldwide.

Obesity complications refer to a range of neurological disorders (e.g., cognitive decline, neuroinflammation), behavioral alterations, and metabolic complications (e.g., type 2 diabetes, metabolic dysfunction-associated steatotic liver disease). Obesity exerts detrimental effects on the brain through multiple mechanisms, such as alterations in brain structure, neuroinflammation, and disrupted energy metabolism. These changes may contribute to the acceleration of obesity complications and further induce cognitive impairment. For example, Wu et al. reported that upregulation of hypothalamic liver kinase B1 alleviates hypothalamic inflammation and protects the melanocortin system from inflammation-induced damage, thereby reducing food intake and fat accumulation and ultimately protecting the development of obesity in mice [2]. In addition, Grasso reported that obesity-induced leptin resistance impairs leptin’s ability to regulate energy balance and the metabolism. This dysfunction affects key brain regions, including the hippocampus, further triggering neuroinflammation and compromising the blood–brain barrier (BBB), which ultimately exacerbates obesity-related cognitive impairment [3].

Polyphenols, a broad category of secondary plant metabolites, are commonly observed in tea, fruits, cereals, vegetables, coffee, olives and wine [4]. Structurally, polyphenols contain one or more phenolic hydroxyl groups and commonly exist in glycosylated forms. They are commonly grouped into four categories according to their phenolic rings and structural linkages: phenolic acids, flavonoids, stilbenes and lignans. The Mediterranean diet, widely recognized as a healthy dietary pattern, is characterized by a high intake of polyphenols that may underlie many of its protective effects. Polyphenol-rich diets are associated with strong antioxidant and anti-inflammatory benefits. Emerging research indicates that regular polyphenol consumption is associated with a reduced risk of metabolic disorders such as diabetes, obesity, chronic inflammation and neurological impairment [5,6,7]. The protective role of polyphenols against metabolic syndrome has also been well-demonstrated. Given this evidence, we hypothesize that polyphenols hold considerable potential to mitigate obesity complications through their neuroregulatory actions. This review systematically summarizes recent advances in the interaction between obesity and brain mechanisms and highlights which polyphenolic compounds contribute to the prevention and treatment of obesity-related neurological disorders from the perspectives of appetite regulation, the gut–brain axis, central neuroinflammation, brain leptin and insulin resistance, and cognitive improvement.

## 2. The Interaction Between Obesity and the Brain

Obesity occurs due to an imbalance between energy intake and energy expenditure.

When caloric intake chronically exceeds energy consumption, the surplus energy accumulates in the body, ultimately leading to obesity. Unhealthy dietary patterns and physical inactivity are the most prominent behavioral contributors to this condition [8].

Importantly, the relationship between obesity and the brain is bidirectional, involving intricate mechanisms such as appetite and energy balance, endocrine regulation, inflammatory responses, neurotransmitter signaling, and the gut–brain interactions.

### 2.1. Brain Regulating Appetite and Energy Balance

#### 2.1.1. Role of the Hypothalamus in Appetite and Energy Regulation

The hypothalamus serves as a primary neural center for energy homeostasis and consists of multiple nuclei that integrate peripheral and central metabolic signals. Among these, the arcuate nucleus (ARC) hosts two key populations of first-order neurons: Agouti-related peptide (AgRP) neurons and pro-opiomelanocortin (POMC) neurons. Located near the third ventricle and median eminence, these neurons can directly sense circulating hormones, such as leptin secreted from adipocytes and insulin produced by pancreatic cells [9], thereby regulating the energy balance in response to circulating nutritional cues [10]. Specifically, AgRP neurons, activated under fasting conditions, drive feeding behavior by releasing the AgRP, neuropeptide Y (NPY), and γ-aminobutyric acid (GABA). In contrast, POMC neurons are stimulated under energy-sufficient states and secrete the α-melanocyte-stimulating hormone (α-MSH), which triggers the melanocortin 4 receptor, leading to downstream signaling that induces satiety, suppresses appetite, and enhances energy expenditure [11]. Beyond feeding behavior, the hypothalamus is integral to regulating thermogenesis and energy metabolism in brown adipose tissue (BAT). Evidence indicates that POMC neurons stimulate BAT thermogenesis through α-MSH-mediated pathways that involve the paraventricular nucleus (PVN) and dorsomedial hypothalamus neurons [12]. Conversely, ARC AgRP and NPY neurons suppress BAT thermogenesis by inhibiting tyrosine hydroxylase-expressing PVN neurons through NPY-mediated signaling [13]. In obesity, AgRP neurons often display persistent hyperexcitability, which drives excessive food intake and further weight gain [14]. In addition to neuronal populations, non-neuronal cell types in the hypothalamus are also essential for the regulation of neural circuits that control energy regulation. Astrocytes can sense metabolic hormones, such as insulin and leptin [15,16]; modulate AgRP activity; and promote gliosis, which enhance neuronal excitability. Similarly, microglia and oligodendrocytes contribute to shaping hypothalamic circuits. However, in the obesity state, these regulatory processes are often disrupted, further impairing hypothalamic function [17,18]. Moreover, the suprachiasmatic nucleus of the hypothalamus functions as the primary circadian regulator of the body, coordinating systemic metabolism and energy homeostasis. By aligning physiological functions with external light–dark cycles and nutrient availability, circadian rhythms ensure metabolic balance. However, disruption of circadian rhythms is a key contributor to impaired insulin sensitivity and disturbances in glucose and lipid homeostasis and further development of obesity complications.

#### 2.1.2. Other Brain Regions in Appetite and Energy Regulation

Other brain regions beyond the hypothalamus also participate in appetite and energy regulation. Among them, the brainstem is particularly important, as it contains multiple nuclei involved in this regulation. Specifically, the dorsal vagal complex, composed of the area postrema, the nucleus tractus solitarius (NTS), and the dorsal motor nucleus of the vagus nerve, serves as a major hub that receives homeostatic inputs from peripheral mediators and relays them to higher brain regions to coordinate energy balance [19].

Additionally, the amygdala and hippocampus contribute to the interplay between emotion, memory, and dietary behavior. The amygdala is associated with emotional responses to food, such as a preference for sweet tastes, and interfaces with the brain’s reward pathways to enhance the hedonic value of eating. The hippocampus, by storing and retrieving food-related memories, interacts with the dopaminergic system to intensify food-related cravings when individuals encounter food cues.

### 2.2. Obesity-Induced Structure and Function Alterations of the Brain

Obesity affects the brain through multiple mechanisms, most notably by inducing neuroinflammation and insulin/leptin resistance. These processes subsequently alter brain structure, function, and metabolism, ultimately leading to cognitive decline and impaired appetite regulation.

#### 2.2.1. Obesity-Induced Brain Structural Changes

Accumulating evidence highlights a strong association between obesity and structural alterations of the brain [20]. Obesity is consistently linked to brain atrophy, which is characterized by gray matter loss, reduced cortical thickness and compromised white-matter integrity [21]. Meta-analysis findings suggest that elevated body mass index (BMI) is strongly correlated with reduced total brain volume. Interestingly, waist circumference and waist-to-hip ratio showed no significant correlation with total brain volume, suggesting that generalized obesity is more strongly linked to brain volume reduction than central obesity [22]. Individuals with obesity have shown reduced gray matter volume in regions governing cognitive control, reward processing, and emotional regulation [23]. Moreover, impaired white-matter integrity has been observed in individuals with elevated BMIs [24]. Over the past decade, epidemiological studies and intervention trials utilizing magnetic resonance imaging have provided evidence that obesity correlates with changes in brain structure and function [25]. The results suggested that addressing obesity-related brain injury via bariatric surgery or dietary intervention may support the maintenance of brain integrity and cognitive performance.

#### 2.2.2. Obesity-Induced Neuroinflammation

Obesity is widely regarded as a state of chronic low-grade inflammation. Excessive lipid accumulation in adipose tissue stimulates immune cell activation, resulting in elevated levels of key inflammatory cytokines, including tumor necrosis factor-alpha (TNF-α), interleukin-1 beta (IL-1β) and interleukin-6 (IL-6). They are capable of crossing a compromised BBB via cellular, humoral and neural pathways, where they disrupt brain neurotransmitter metabolism, neuroendocrine signaling, synaptic plasticity and motor neuron transmission. These lead to widespread neuroinflammation and functional impairments in the nervous system. Neuroinflammation is not only regarded as a key factor for metabolic syndrome but also for cognitive impairment [26]. Obesity-related inflammation in the hypothalamus is marked by increased pro-inflammatory markers and enhanced activity of glial cells, including astrocytes and microglia [27,28,29]. As the primary immune cells in the brain, microglia express Toll-like receptor 4 (TLR4), which recognizes long-chain fatty acids. In a diet-induced obesity model, Moser et al. reported that the TLR4 inhibitor TAK-242 suppressed TNF-α and IL-6 production in the hypothalamus. It also attenuated high-fat diet (HFD)-induced neuronal damage and improved the function of neural networks related to energy homeostasis, which highlights the central role of TLR4 in obesity-related neuroinflammation [30]. In animal models of obesity, activation of Toll-like receptor signaling by dietary lipids drives the production of pro-inflammatory cytokines (TNF-α, IL-1β, and IL-6), which subsequently activate downstream pathways, including the c-Jun *N*-terminal kinase (JNK) [31] and IκB kinase β/nuclear factor kappa B (NF-κB) pathways [32]. This signaling cascade propagates neuroinflammation and promotes hypothalamic gliosis, contributing to central leptin and insulin resistance. Consequently, neuronal circuits controlling energy homeostasis are impaired, resulting in dysregulated appetite and energy expenditure [33]. In addition, obesity-induced chronic inflammation and HFDs can compromise the integrity of the BBB, thereby enhancing its permeability and enabling peripheral inflammatory cytokines to infiltrate the brain, further exacerbating neuroinflammation.

Importantly, these effects extend beyond the hypothalamus to actually affect the entire brain’s regions [34]. Prolonged HFD feeding induces inflammation in the brain regions involved in mood and cognition, including the prefrontal cortex, amygdala, and hippocampus [9].

#### 2.2.3. Obesity-Induced Brain Insulin and Leptin Resistance

The critical function of insulin in body weight and appetite was first highlighted in the late 1970s, when Stephen Woods and his colleagues reported decreased food intake and body weight in baboons as a result of prolonged low-dose central insulin infusion [35]. This finding emphasized the importance of insulin in the regulation of appetite and body weight within the brain. Insulin signals through its receptors to initiate downstream signaling pathways in he brain. Selective removal of insulin receptors in neurons leads to obesity, characterized by increased adiposity and hepatic insulin resistance in mice [36], underscoring the significance of brain insulin signaling in systemic glucose regulation. Beyond metabolic regulation, insulin plays a direct role in neurotransmission and synaptic plasticity. It is highly expressed at the synapses, where it modulates ion channels including GABA, *N*-methyl-D-aspartate (NMDA), and α-amino-3-hydroxy-5-methyl-4-isoxazolepropionic acid (AMPA) receptors, thereby shaping neurotransmission and synaptic plasticity [37,38]. Neuroimaging studies in humans have further suggested that central insulin signaling modulates region-specific neural activity and functional connectivity patterns, which in turn impact cognition, feeding behavior, and metabolism [39].

Brain insulin resistance is a shared neuropathological feature of obesity and dementia and is also considered a major contributor to metabolic and cognitive dysfunction [40]. Several mechanisms link obesity to central insulin resistance. One of these mechanisms involves the accumulation of harmful free fatty acids in the brain as a result of HFD intake. Elevated circulating free fatty acids during HFD intake induce ceramide accumulation in the brain. These ceramides trigger inflammation through activation of the NF-κB pathway, which promotes the release of inflammatory cytokines such as TNFα, IL-1β, and IL-6, which impair insulin signaling and activate JNK, contributing to endoplasmic reticulum stress and further disrupting brain insulin-signaling pathways [41]. Oxidative stress may serve as an additional mediator linking obesity to insulin resistance. Obesity increases reactive oxygen species levels in the brain and impairs nuclear factor erythroid 2-related factor 2 (Nrf2)-driven antioxidant responses. The resulting oxidative stress interferes with insulin signaling by disrupting proteins such as insulin receptor substrate 1 (IRS-1), thereby exacerbating central insulin resistance.

Leptin is mainly secreted by adipocytes, and its circulating concentration typically increases with greater body fat stores. Within the brain, the hypothalamus represents a principal site where leptin exerts its regulatory effects on energy metabolism. Upon reaching the ARC of the hypothalamus, leptin binds to its receptor, OB-Rb, to activate downstream signaling pathways and induce the signal transducer and activator of transcription 3 (STAT3) phosphorylation in appetite-regulating neurons, thereby suppressing appetite and enhancing energy expenditure. In addition, leptin acts through the AMP-activated protein kinase (AMPK) signaling pathway to activate acetyl-CoA carboxylase (ACC), exerting anorexigenic effects in both the ARC and PVN [42].

Most obese individuals develop leptin resistance, a condition marked by a reduced response to elevated circulating leptin (hyperleptinemia) within the brain. This impaired signaling results in diminished appetite suppression and reduced energy expenditure [43]. Several potential mechanisms underlying obesity-associated leptin resistance have been proposed: (1) reduced efficiency of leptin transport across the blood–brain barrier, preventing circulating leptin from reaching its neuronal targets [44]; (2) impaired Ob-Rb function or inhibition of downstream signaling, resulting in insufficient STAT3 phosphorylation and failure to activate anorexigenic pathways; (3) overactivation of negative feedback regulators, such as suppressor of cytokine signaling 3 (SOCS3) and protein tyrosine phosphatase 1B (PTP1B), which block leptin signaling; and (4) HFD-induced hypothalamic inflammation disrupting leptin signaling pathways. Collectively, these factors contribute to impairments of leptin signaling, and weaken the leptin regulation of appetite and energy balance.

#### 2.2.4. Obesity-Induced Cognitive Dysfunction

Cognitive dysfunction is a common complication of obesity and has been implicated in the development of Alzheimer’s disease (AD) [45,46]. Epidemiological studies have shown that abdominal obesity increases the risk of cognitive impairment [47]. However, the relationship is not linear and varies with age. An integrated analysis of large population datasets revealed a positive association between midlife obesity and dementia, whereas a negative association was observed in late life [48], with the adverse effects of obesity on cognition being more pronounced in men. Another report also indicated that late-life obesity was linked to improved attention and executive function performance [49]. This evidence points to a significant U-shaped association between obesity and dementia risk.

**Figure 1:**
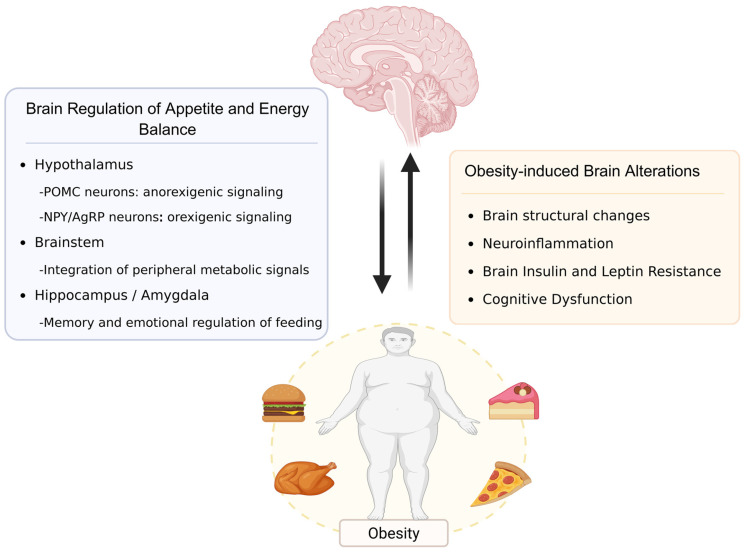
The interaction between obesity and the brain. Key brain regions, including the hypothalamus, brainstem, hippocampus, and amygdala, regulate appetite and maintain energy balance. In obesity, excessive energy intake is associated with brain structural changes, neuroinflammation, insulin and leptin resistance, and cognitive dysfunction. Created in https://BioRender.com.

Obesity elevates the risk of cognitive impairment through multiple mechanisms. Synaptic plasticity is the fundamental basis of cognitive function and represents a key mechanism underlying learning and memory in the nervous system [50]. Molecules including synapsin and postsynaptic density protein 95 (PSD95) are crucial for synapse formation and the maintenance of synaptic plasticity. Compared with control groups, diet-induced-obese mice exhibited markedly decreased hippocampal expression of synapsin and PSD95, disruption of hippocampal synaptic ultrastructure, and consequent cognitive decline [51]. The brain-derived neurotrophic factor (BDNF) participates in mechanisms that support synaptic plasticity and neuronal survival, functioning by interacting with its cell surface receptor, tropomyosin receptor kinase B. This interaction triggers activation processes that maintain the healthy functioning of the nervous system, promote synaptic remodeling, support neuronal survival, and optimize neural function through the activation of various intracellular signaling pathways, such as PI3K/Akt, Ras/MAPK, and PLC signaling. Obesity has been shown to reduce BDNF expression, thereby impairing neuroplasticity [52]. Beyond these deficits, obesity impairs cognition through synergistic mechanisms including chronic neuroinflammation, insulin resistance, and oxidative stress, along with disturbances in the gut–brain axis, which cumulatively contribute to cognitive impairment. The interaction between obesity and the brain is illustrated in Figure 1.

### 2.3. Gut–Brain Axis Regulates Energy Homeostasis

As an important communication network between the gastrointestinal tract and the brain, the gut–brain axis functions as a key regulator of maintaining metabolic balance and energy homeostasis through neural, endocrine, and immune signaling. For example, microbial metabolites derived from the fermentation of dietary fiber can be sensed by enterochromaffin and enteroendocrine cells in the gut [53], which stimulates the secretion of gut hormones such as GLP-1 and PYY. These signals can reach the brain through vagal afferent pathways or the circulatory system. Conceptually, this unidirectional flow can be summarized as a functional signaling framework: gut microbiota → microbial metabolites (e.g., short-chain fatty acids) → gut hormone secretion → neural or humoral transmission → central regulation. Gut–brain communication also occurs through interactions between gut microbes and intestinal immune cells, which can exert local effects in the gut, stimulate vagal afferent terminals, or trigger systemic immune responses (e.g., metabolic endotoxemia). This systemic response can ultimately contribute to neuroinflammation by affecting other organs and target cells, including brain glial cells [54]. The hypothalamus serves as a key regulatory center in the brain, integrating these gut-derived signals with other metabolic inputs to coordinate food intake, energy expenditure, and glucose homeostasis [55].

#### 2.3.1. Gut Microbiota Regulate Energy Homeostasis

A diverse microbial community inhabits the gut, including bacteria, archaea, viruses, and protozoa, which are essential for maintaining physiological functions in both healthy and diseased conditions. Its composition is closely associated with obesity. Fecal microbiota transplantation suggests a causal role of the gut microbiome in metabolic disorders [56]. Dietary patterns represent a primary determinant of gut microbial composition. Growing evidence indicates that *Prevotella* abundance is associated with diets rich in carbohydrates and high-glycemic-index foods, whereas *Bacteroides* is linked to long-term intake of dietary protein and animal fat [57]. Comparative studies indicate a significantly higher abundance of Firmicutes and a marked reduction in Bacteroidetes in individuals with obesity compared with healthy individuals [58]. Interestingly, a one-year low-fat, low-carbohydrate diet reversed these proportions, causing a marked decrease in body weight, further supporting the role of the gut microbiota in host energy regulation. Nutrient-induced growth of *Escherichia coli* (*E. coli*) in mice increased plasma peptide YY (PYY) and glucagon-like peptide-1 (GLP-1) levels and activated POMC neurons, suggesting that specific microbes may signal the termination of feeding [59].

In obesity complications, gut microbiota undergo dysbiosis [60], often characterized by an increased abundance of energy-harvesting microbial species. This shift disrupts systemic homeostasis and central appetite regulation, creating a vicious cycle that exacerbates obesity and metabolic dysfunction. Furthermore, dysbiosis can impair intestinal epithelial tight junctions, increasing intestinal permeability, and allowing endotoxins to enter the circulation [61]. This process triggers inflammation in both the gut and the brain, which further amplifies obesity-related neuroinflammation and metabolic disorders [62].

#### 2.3.2. SCFAs Regulate Energy Homeostasis

SCFAs are metabolites produced through microbial fermentation of dietary fiber in the distal small intestine and colon [63]. Acetate, propionate and butyrate represent the predominant SCFAs in humans and rodents, accounting for approximately 95% of total SCFAs. These molecules act as ligands for free fatty acid receptor 2 (FFAR2, also known as GPR43) and free fatty acid receptor 3 (FFAR3, also known as GPR41). Acetate exhibits a higher affinity for FFAR2 [64], while propionate and butyrate are potent agonists for FFAR3. In addition, butyrate acts as the major energy substrate for colonic epithelial cells and is a key microbial metabolite influencing host physiology [65].

The SCFAs contribute to the energy balance either by activating vagus nerve-mediated appetite-signaling pathways or by reaching the brain through systemic circulation to stimulate the release of gut–brain peptides [66]. In mice, acute oral administration of butyrate, but not intravenous infusion, significantly reduced food intake, inhibited the activity of hypothalamic NPY-expressing orexigenic neurons, and suppressed neural activity within the NTS and dorsal vagal complex of the brainstem. Long-term supplementation with butyrate has been demonstrated to prevent diet-induced obesity and hepatic steatosis primarily by decreasing food consumption [67]. Conversely, HFD intake can alter the gut microbiota to decrease acetate production, which, in turn, activates the parasympathetic nervous system to promote insulin and ghrelin secretion and may contribute to hyperphagia and obesity [68]. However, acetate has also been demonstrated to cross the BBB and induce anorexigenic signaling in the hypothalamus [69], suggesting a potential appetite-suppressing effect. In addition, the expression of SCFA transporters and receptors in brain tissue indicates that SCFAs may also exert a direct effect on the brain.

#### 2.3.3. Intestinal and Brain Hormones Regulate Food Intake

Appetite regulation and the perception of food intake are modulated by many hormones secreted primarily from stomach and intestines. These hormones influence energy metabolism and feeding behavior by acting on specific receptors within the brain and peripheral tissues. This section examines the roles of three key hormones, including PYY, GLP-1, and cholecystokinin (CCK).

PYY is a gut-derived peptide, composed of 36 amino acids, that is released from intestinal L-cells in response to nutrient intake and belongs to the pancreatic polypeptide family. Its primary endogenous forms are PYY1-36 and PYY3-36. PYY3-36 is a predominant circulating form postprandially. Circulating PYY levels are at their peak 1−2 h post-meal and stay elevated for hours, indicating PYY’s role in sustaining long-term satiety. Batterham et al. reported that PYY3-36 infusion reduced food intake by 36% within 90 min in individuals with obesity [70]. PYY is known to inhibit food intake by binding to Y1 and Y2 receptor subtypes in rodents through stimulating POMC neurons. Its anorexigenic effect is primarily mediated through the Y2 receptors, which are widely expressed in the NPY neurons of the ARC. Stimulation of Y2 receptors inhibits orexigenic neuronal activity and reduces NPY expression [71]. PYY also stimulates POMC neurons, although it can induce satiety independent of the POMC pathway [72]. Furthermore, PYY3-36 activates fos expression in key appetite-regulating brain regions, including the ARC and the area postrema in rodents [73].

GLP-1, derived from the preproglucagon gene, is released from enteroendocrine L cells located in the distal intestine and colon following nutrient ingestion. This hormone effectively inhibits gastric acid secretion, delays gastric emptying, and promotes satiety. Upon release into bloodstream, GLP-1 has a very short plasma half-life of approximately two minutes, as it is quickly inactivated by dipeptidyl peptidase IV. Intracerebroventricular administration of the GLP-1 receptor (GLP-1R) agonist exendin-4 significantly reduced food intake and body weight in rats, accompanied by reduced levels of plasma-active ghrelin in the plasma, the hypothalamus, and the stomach [74]. On the other hand, peripheral treatment with the GLP-1R agonists exendin-4 and liraglutide also inhibits food intake, an effect mediated by activating GLP-1Rs on vagal afferent neurons and through direct activation of the central GLP-1R, highlighting the importance of interplay between central and peripheral GLP-1 signaling [75]. Preproglucagon neurons in the NTS represent an important source of endogenous central GLP-1 [76]. Knockdown of the preproglucagon gene in the NTS has been shown to promote excessive food intake and body weight gain, indicating a key role of central GLP-1 in appetite regulation [77]. However, recent findings propose that the peripheral and central GLP-1 systems may function separately, as vagal afferent fibers expressing GLP-1R provide minimal innervation to NTS preproglucagon neurons [78].

CCK was one of the earliest gut hormones identified to exert anorexigenic effects. It is secreted mainly by type I enteroendocrine cells in the mucosa of the small intestine in response to intestinal fatty acids and proteins. CCK is also widely distributed throughout the gastrointestinal tract and nervous system and exists in several biologically active forms, with CCK-8, CCK-33, and CCK-58 being the most common [79]. It plays important roles in regulating gastrointestinal function and energy metabolic homeostasis by acting on CCK receptors [80]. Two receptor subtypes of CCK have been characterized: CCK-1R and CCK-2R. Exogenous administration of CCK-8 has been shown to activate receptors expressed on vagal afferent neurons, which in turn stimulate specific neuronal populations in the brainstem and project to the PVN of the hypothalamus, thereby suppressing appetite [81]. In addition, postprandial plasma levels of CCK will increase significantly and can reach the brain via circulation, where CCK directly acts on central targets to reduce food intake.

The gut microbiota are involved in the regulation of gut hormone secretion. Gut microbes produce various metabolites that directly activate intestinal enteroendocrine cells [82]. Through the microbiota–gut–brain axis, gut bacteria can affect brain activity by regulating endocrine pathways involving GLP-1 and PYY, thereby participating in appetite and energy control [83]. For example, the microbial metabolite butyrate promotes leptin secretion from adipocytes and stimulates GLP-1 release from L-cells to precipitate energy homeostasis. Mice fed HFDs exhibited altered gastrointestinal hormone secretion [84] and impaired vagal afferent sensitivity [85], which disrupted appetite regulation and metabolic homeostasis. In obesity-related pathological states, gut–brain communication is substantially disrupted. Impaired neuronal sensitivity to anorexigenic hormones, such as CCK, GLP-1, and PYY, may exacerbate obesity complications [86].

## 3. Dietary Polyphenols Regulate Nervous System to Ameliorate Obesity Complications

### 3.1. Polyphenols Ameliorate Obesity by Regulating Appetite and Feeding Patterns

Polyphenols may exert anti-obesity effects by suppressing appetite through modulation of the central neural pathways. Research has found that (−)-epigallocatechin-3-gallate (EGCG), the most abundant polyphenol in green tea, can cross the BBB and reach the brain parenchyma [87] and exhibits its anti-obesity effects by reducing appetite. Animal studies have provided initial evidence for reducing appetite through EGCG action. Intraperitoneal injection of EGCG suppressed appetite in both lean and obese male Zucker rats, indicating that its effects are not reliant on leptin receptors and may involve a leptin-independent mechanism for appetite regulation [88]. Notably, this anorexigenic effect was not observed with other catechins (e.g., (−)-Epicatechin, (−)-Epigallocatechin, or (−)-Epicatechin-3-gallate) under comparable conditions. Furthermore, in mice, the co-administration of 0.1% EGCG and 0.1% caffeine induced a marked anorexigenic effect, potentially by delaying GLP-1 induced gastric emptying and increasing hypothalamic POMC expression, a crucial factor in appetite regulation [89]. Evidence from two human studies may further support the appetite-regulating effects of EGCG. Acute EGCG supplementation (752 mg) delayed gastric emptying and enhanced satiety at 90 min in 22 healthy women over a 150 min study period, while there were no significant differences in glucose, insulin, and leptin concentrations between the treated and untreated groups [90]. Another randomized crossover trial involving 36 healthy men showed that acute ingestion of green tea catechins (540 mg) combined with chlorogenic acids over a 240 min postprandial period increased GLP-1 secretion and reduced glycemic responses [91]. These two studies both involved small sample sizes (22 vs. 36 participants) and short-term observations (150 min vs. 240 min), which provided preliminary evidence for the potential of EGCG in appetite regulation and GLP-1 modulation. However, the findings are still limited by the short observation periods and relatively small sample sizes. Further large-scale and long-term human studies are needed to clarify the effects of EGCG on GLP-1 secretion and appetite regulation.

Polyphenols modulate appetite through multiple pathways. Chlorogenic acid has been shown to suppress orexigenic genes (*Agrp*, *Npy*) and upregulate anorexigenic gene (*Pomc*, *Cartpt*) expression in HFD-induced rat models, indicating its role in appetite via direct and indirect mechanisms [92]. Similarly, apple polyphenol extracts have also been shown to activate enteroendocrine cells to release GLP-1 and subsequently upregulate hypothalamic anorexigenic genes such as *Pomc*, *Cart*, and *Mc4r*, thereby enhancing satiety, decreasing food intake, and ultimately resulting in weight loss [93].

Hypothalamic neurogenesis is thought to be crucial for energy homeostasis. HFDs have been shown to disrupt the equilibrium between orexigenic and anorexigenic neuronal populations in the hypothalamus. Resveratrol, a stilbene-type polyphenol, is present in dietary sources like red grape skins and berries. Studies have shown that resveratrol supplementation in HFD-fed animals enhances the generation of new cells across multiple hypothalamic regions, specifically in the ARC. HFDs alone promote the differentiation of newborn cells primarily into NPY neurons. However, the addition of resveratrol reverses this trend, favoring differentiation toward POMC neurons. These findings suggest that resveratrol may help regulate body weight by modulating hypothalamic neurogenesis and appetite control [94]. Further investigations have indicated that resveratrol not only reduces body weight and fat accumulation while enhancing the generation of anorexigenic neurons [95] but also dose-dependently suppresses NPY and AgRP expression. This effect was associated with a marked reduction in food intake, indicating a clear dose-dependent regulatory effect of resveratrol on orexigenic neuropeptides [96].

Persistent disruption of feeding patterns can interfere with the circadian rhythm of the body, ultimately leading to metabolic dysfunction and obesity. HFDs contribute to obesity through excessive caloric intake and disruption of normal feeding rhythms. Dietary polyphenols have demonstrated remarkable effects in ameliorating disordered feeding behaviors. By quantifying total food intake, meal size, and feeding frequency, the results have shown that EGCG treatment effectively suppressed daytime binge eating associated with HFD feeding. Concurrently, 0.5% (*w*/*w*) EGCG supplementation altered the diurnal oscillatory expression patterns of key appetite-regulating genes (such as *Agrp*, *Pomc*, and *Cart*) and core circadian clock genes (*Clock* and *Bmal1*) in the hypothalamus of diet-induced-obese mice, highlighting its critical role in regulating feeding behavior and maintaining energy homeostasis [97]. In addition, polyphenols extracted from black soybean seed coats significantly attenuated hypothalamic microglial activation in HFD-fed mice and normalized feeding behavior, ultimately improving diet-induced obesity [98].

### 3.2. Polyphenols Prevent Obesity by Inhibiting Central Inflammation

Neuroinflammation contributes to neuronal network dysfunction, neuronal injury, and even neuron cell death, thereby accelerating the onset and progression of obesity complications. EGCG has demonstrated potent anti-inflammatory effects not only in peripheral tissues but also within the brain. EGCG may alleviate HFD-induced obesity by modulating hypothalamic inflammation. Dietary supplementation with 1% EGCG markedly suppressed the phosphorylation of key proteins in the NF-κB and JAK2/STAT3 pathways in the hypothalami of mice with HFD-induced obesity. Consequently, levels of pro-inflammatory cytokines (TNF-α, IL-6, and IL-1β) were significantly decreased, leading to reduced hypothalamic inflammation and neuronal injury [6]. Mi et al. reported that EGCG (255 mg/kg/d) alleviated neuronal damage and cognitive dysfunction associated with diet-induced metabolic disturbance by inhibition of the MAPK and NF-κB pathways and downregulating inflammatory mediators like TNF-α [99]. In addition, Mao et al. reported that EGCG suppressed lipid accumulation, pro-inflammatory cytokine release, and microglial activation in both palmitic acid-stimulated microglial cells and HFD-induced-obese mice. The underlying mechanism was primarily mediated through inhibition of the JAK2/STAT3 signaling pathway in the microglia, ultimately attenuating hypothalamic inflammation associated with HFD exposure [100]. Based on pharmaceutical scaling calculations, 255 mg/kg/day in mice is equivalent to approximately 5 cups of green tea per day in humans. This is achievable dosage for the casual green tea consumer. However, 1% (*w*/*w*) EGCG supplementation corresponds to approximately 15.6 cups of green tea per day. This is very higher amount of EGCG than that daily intake of the human consumer. Notably, the biological effects of EGCG are dose-dependent. However, the exposure to supraphysiological doses (1000 mg/kg) has been linked to negative outcomes [101].

Resveratrol is known to suppress the activation of glial cells and decrease the production of pro-inflammatory cytokines [102]. Peroxisome proliferator-activated receptor gamma coactivator-1 alpha (PGC-1α) was reported to suppress M1 polarization of microglia triggered by LPS through inhibition of NF-κB signaling while promoting M2 polarization through activation of the STAT6 and STAT3 pathways. During neuroinflammation, PGC-1α expression is often downregulated. However, resveratrol has been reported to upregulate PGC-1α expression, thereby alleviating inflammatory damage and driving the microglia to adopt an anti-inflammatory M2 phenotype [103]. Another study revealed that HFD feeding impairs mitochondrial fission by suppressing the Sirtuin 1 (SIRT1)/PGC-1α signaling pathway, which leads to NLRP3 inflammasome activation, increased oxidative stress, and neuronal pyroptosis, ultimately contributing to obesity-related cognitive impairment. Resveratrol (120 mg/kg/day) activates the SIRT1/PGC-1α pathway, restores mitochondrial dynamics, and consequently ameliorates HFD-induced neuroinflammation and neuronal damage, highlighting its neuroprotective potential and therapeutic relevance [104]. In addition to its effects on PGC-1α, recent findings suggest that resveratrol may activate the AMPK/ACC signaling pathway, which enhances insulin sensitivity and alleviates neuroinflammation, thereby improving cognitive dysfunction in HFD-fed mice [105].

Anthocyanins are a subclass of flavonoids and represent a group of naturally occurring pigments widely distributed in plants. Anthocyanin-rich extracts provide notable neuroprotective and anti-inflammatory benefits. Tart cherry (*Prunus cerasus*), a fruit rich in anthocyanins, reduces glial fibrillary acidic protein expression and microglial activation while increasing neurofilament levels in the hippocampus and prefrontal cortex of diet-induced-obese rats. Tart cherry intake improved the expression of aquaporin-4 and endothelial inflammatory markers. These findings suggest that tart cherry may help prevent obesity by attenuating neuroinflammation [106]. Juçara, a palm fruit native to the Atlantic Forest, is also rich in anthocyanins. Research reported that dietary supplementation with 0.25% Juçara exerts anti-neuroinflammatory effects by inhibiting the activation of the NF-κB signaling pathway induced by an HFD [107]. A dietary supplement enriched with cyanidin and delphinidin also attenuated hippocampal inflammation, microglial proliferation, and reductions in mineralocorticoid receptor and BDNF expression in HFD-fed mice, thereby mitigating obesity-related neurological inflammation [108]. Additionally, anthocyanin-rich blackberry extract demonstrated robust anti-inflammatory activity in the brain [109].

Quercetin is a natural flavanol extracted from fruits and plants. It has been demonstrated that quercetin alleviates hypothalamic inflammatory responses in obese mice by inhibiting inflammation driven by microglial activation through the induction of heme oxygenase-1 (HO-1) [110]. HO-1 functions as a key antioxidant enzyme involved in cellular defense against oxidative damage and inflammation. Curcumin is the primary bioactive compound in turmeric rhizomes. Curcumin offers multiple potential health benefits for metabolic syndrome and neuroprotection through anti-inflammatory effects [111]. Studies have shown that upregulation of FKN/CX3CR1 contributes to neuroinflammation in mice fed fructose. Curcumin inhibits this upregulation, thereby preventing neuronal injury and highlighting its potential as a dietary supplement for managing diet-induced neuroinflammation and obesity [112].

### 3.3. Polyphenols Ameliorate Obesity by Enhancing Brain Insulin/Leptin Sensitivity

Central insulin resistance is closely associated with obesity complications. Dietary polyphenols, particularly resveratrol, have shown significant effects in ameliorating HFD-induced obesity by improving brain insulin resistance. Although resveratrol is barely detectable in peripheral tissues following intragastric delivery, it accumulates in the intestinal mucosa at concentrations substantially higher than in serum. In rat models, acute intraduodenal infusion (60 ng/min for 50 min) of resveratrol activated SIRT1 and AMPK in the duodenum, thereby initiating the gut–brain–liver neural axis, enhancing hypothalamic insulin sensitivity, and subsequently suppressing hepatic gluconeogenesis and obesity complications [113]. The results indicated that local intestinal actions may partially compensate for limited systemic bioavailability. Given its extremely low oral bioavailability (<1%), the metabolic effects of resveratrol may partly depend on locally initiated intestinal signaling. Nevertheless, the majority of studies employ doses of resveratrol that are considerably higher than levels achievable through dietary intake. Recent advances in nanoformulation approaches, such as brain-targeted peptide-functionalized chitosan nanoparticles for resveratrol delivery, have demonstrated enhanced tissue targeting, improved stability, and superior metabolic efficacy compared to free resveratrol. Such delivery systems may overcome pharmacokinetic limitations, enhance bioavailability, and facilitate translational application. Furthermore, brain-targeted delivery of resveratrol nanoparticles enhances obesity-related glucose homeostasis by inhibiting insulin resistance caused by lipid accumulation through the JNK/AKT/GSK3β signaling pathway [114]. EGCG also shows excellent efficacy in improving brain insulin resistance. A high-fat, high-fructose diet (HFFD) impairs insulin signaling in the mouse brain, whereas EGCG has been shown to restore this signaling by activating the IRS-1/AKT pathway. In vitro studies using SH-SY5Y neuronal cells further indicate that EGCG attenuates insulin resistance caused by high glucose, supporting its neuroprotective properties [99]. In addition, cinnamon, which is rich in polyphenolic compounds, effectively mitigates HFFD-associated insulin resistance by regulating the PI3K-AKT-GSK3β signaling pathway [115]. Moreover, studies have indicated that purple sweet potato color (PSPC), a natural anthocyanin, ameliorates impaired insulin signaling in the hippocampi of mice exposed to HFDs by upregulating the expressions of p-IRS1, PI3K p110α, and p-AKT. PSPC also significantly downregulates the obesity-induced abnormal elevation of galectin-3 and SOCS3, thereby improving both impaired insulin signaling and inflammation and ultimately alleviating obesity-related insulin resistance [116].

Studies have reported that resveratrol may improve central leptin signaling by reducing leptin secretion and enhancing STAT3 phosphorylation (p-STAT3) in the hypothalamus. These effects contribute to increased leptin sensitivity and are accompanied by downregulation of orexigenic neuropeptides AgRP and NPY [117,118,119]. PTP1B negatively regulates leptin signaling. Polyphenol-rich cherries have enhanced leptin sensitivity in obese rats by significantly suppressing hypothalamic *Ptp1b* mRNA expression under short-day photoperiod conditions in a photoperiod-dependent manner [120]. Chestnut shell polyphenol extract (GSPE) has also been shown to significantly increase leptin levels in mice with obesity and activate the LEPR–JAK2/STAT3–PTP1B–SOCS-3 signaling pathway in hypothalamic tissues, thereby ameliorating leptin resistance [121]. Moreover, GSPE reduces excessive food intake and alleviates both central and peripheral leptin resistance in diet-induced obesity via increased Sirt1 expression and inhibition of hypothalamic inflammation [122]. Seyeon et al. simulated a HFD-induced hypothalamic environment by treating hypothalamic neuronal N1 cells with palmitate and evaluated the effects of four phlorotannins on alleviating leptin resistance. The results showed that treatment with pyrogallol-phloroglucinol-6,6-bieckol significantly attenuated palmitate-induced leptin resistance in hypothalamic neurons, as evidenced by a marked reduction in *Socs3* mRNA levels and significant increases in *Stat3* and *ObR* mRNA expression [123].

### 3.4. Polyphenols Ameliorate Obesity by Regulating the Gut–Brain Axis

Growing evidence suggests that the gut–brain axis may play a pivotal role in preventing obesity, and polyphenols may influence this axis at multiple stages.

Polyphenols may modulate gut microbiota composition and microbial metabolites. Gu et al. revealed that EGCG treatment notably elevated the abundance of the beneficial bacterium *Akkermansia muciniphila* and partially reversed HFD-induced gut microbiota dysbiosis. Additionally, EGCG markedly altered tryptophan metabolism and gene expression in the hypothalamus, thereby modulating gut–brain communication and preventing precocious puberty induced by obesity [124]. Another study by Zhou et al. showed that EGCG reduced colonic inflammatory markers and barrier damage, increased microbial diversity, decreased SCFA levels, and downregulated expression of key transcription factors by oral-gavage EGCG for six weeks using obese male C57BL/6J mice. EGCG was also found to modulate hypothalamic neurotransmitter levels, including dopamine and 5-hydroxytryptophan, indicating that it may mitigate obesity and associated metabolic disturbances through gut–brain interactions [125]. Circadian rhythm is closely associated with the host’s microbiota and obesity. Administration of green tea polyphenols (GTPs) for four weeks significantly improved the composition of the gut microbiota and modulated metabolites related to brain function and circadian rhythm. In parallel, GTP treatment increased the numbers of astrocytes and oligodendrocytes while regulating the expression of core clock genes (*Csnk1d*, *Clock*, *Per3*, *Cry2*, and *Bhlhe41*) disrupted by HFD induction [126].

**Figure 2:**
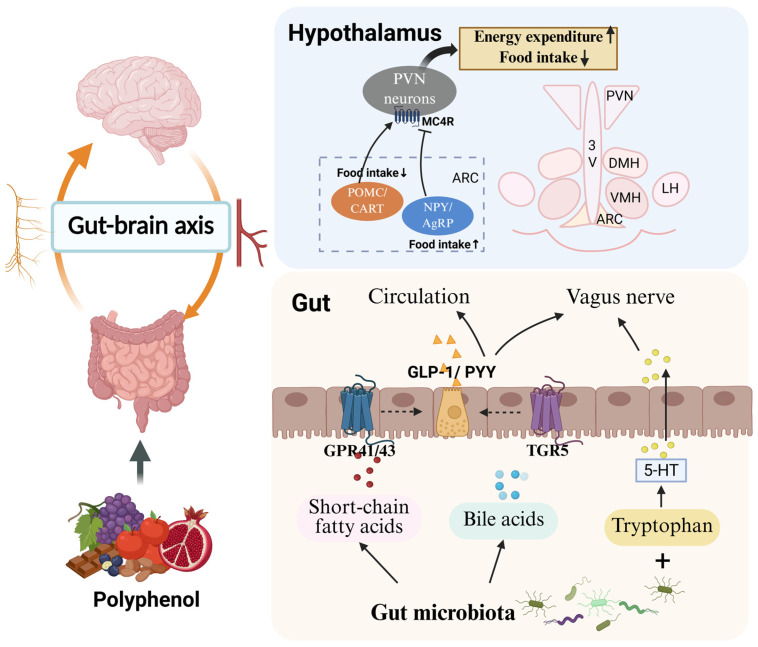
Effects of polyphenols on the gut–brain axis. Polyphenols modulate the gut microbiota and stimulate the production of microbial metabolites, such as short-chain fatty acids and bile acids, which may activate intestinal receptors and promote the secretion of gut hormones including GLP-1 and PYY. Microbiota-related tryptophan metabolism may also contribute to serotonin-associated gut–brain signaling. Together, these signals may influence hypothalamic pathways involved in the regulation of food intake and energy expenditure. Abbreviations: GPR41/43, G protein-coupled receptor 41/43; TGR5, Takeda G protein-coupled receptor 5; 5-HT, 5-hydroxytryptamine; GLP-1, glucagon-like peptide-1; PYY, peptide YY; MC4R, melanocortin 4 receptor; ARC, arcuate nucleus; PVN, paraventricular nucleus; POMC, pro-opiomelanocortin; CART, cocaine- and amphetamine-regulated transcript; NPY, neuropeptide Y; AgRP, Agouti-related peptide. Created in https://BioRender.com.

Polyphenols may regulate the secretion of gut-derived hormones involved in appetite control. EGCG has regulated appetite by stimulating the secretion of gut hormones such as CCK, GLP-1, and PYY [127]. A recent study reported that an EGCG–apo-lactoferrin (Apo-LF) conjugate significantly enhanced GLP-1 secretion in a human colonic cell line (NCI-H716), thereby promoting satiety and underscoring the potential of EGCG as a dietary supplement for prevention of obesity complications [128]. Dysregulated bile acid synthesis, often accompanied by gut microbiota imbalance, contributes to metabolic obesity. EGCG has ameliorated obesity complications by increasing the intestinal abundance of *Akkermansia muciniphila*, enhancing GLP-1 and PYY secretion, activating Takeda G protein-coupled receptor 5 (TGR5), and modulating bile acid signaling [129]. However, these findings are mainly based on in vitro studies and rodent models, and further validation in human clinical trials, which have thus far shown limited effects of EGCG on appetite regulation, is still needed. The effects of polyphenols regulating the gut–brain axis are summarized in Figure 2.

Preclinical evidence suggested that resveratrol may exert anti-obesity effects by modulating the gut–brain axis in a dose-dependent way [130]. Dietary supplementation with resveratrol (200 mg/kg) reduced the body weights of HFD-fed mice, which is associated with improvement in gut microbiota composition, particularly through an increase in SCFA-producing bacteria [131]. Moreover, transplantation of microbiota from resveratrol-treated mice into HFD-fed recipients effectively attenuated body weight increases, improved insulin sensitivity, and promoted lipid metabolic regulation as well as intestinal barrier integrity [132]. Moreover, resveratrol promotes the production of SCFAs by gut microbes, and, further, stimulates multiple receptors located in the gut and brain, initiating beneficial effects that are further transmitted to the brain via the vagus nerve [133]. Recent evidence suggests that anthocyanins prevent obesity complications by counteracting HFD-induced dysbiosis, increasing tryptophan metabolism production and neuroprotective metabolite kynurenic acid [134]. Furthermore, anthocyanin metabolites generated by gut microbes may regulate the secretion of both orexigenic and anorexigenic hormones. For example, delphinidin-3-rutinoside has been demonstrated to enhance GLP-1 secretion from intestinal L-cells, thereby reducing food intake [135]. In addition, cyanidin and delphinidin also promote GLP-1 production in GLUTag cells through activating protein kinase A (PKA)-dependent pathways, which play a crucial role in maintaining gut and glucose homeostasis [136]. A study found that supplementation with cyanidin- and delphinidin-rich extract (CDRE) attenuated metabolic disturbances. However, the effects of CDRE on hormones such as insulin, GLP-1, GLP-2, and GIP were not significantly changed [137]. Therefore, additional studies are required to elucidate the impact of anthocyanins in modulating the gut–brain axis and alleviating obesity complications.

### 3.5. Polyphenols Improve Obesity-Associated Cognitive Impairment

Obesity-related neuroinflammation can disrupt the hypothalamic–pituitary–adrenal axis, impairing stress responses and reducing BDNF expression, which may contribute to anxiety and depression. HFDs induce obesity and dyslipidemia, thereby impairing cholesterol metabolism in the brain, preventing synaptic development, dendritic arborization, and axonal elongation and further leading to β-amyloid (Aβ) accumulation and neuroinflammation, ultimately resulting in cognitive decline. Resveratrol demonstrates promising efficacy in improving HFD-related cognitive dysfunction. Su et al. reported that microbiota-derived resveratrol metabolites significantly enhanced the expression of memory-associated proteins, including phosphorylated CaMKII (p-CaMKII) and synaptophysin, in the brains of HFD-fed mice, indicating its potential to ameliorate synaptic damage and cognitive impairment [138]. Another study reported that resveratrol alleviated cognitive deficits in diabetic rats through activation of SIRT1 and suppression of the TGF-β1 signaling pathway. This SIRT1–TGF-β1 regulatory axis also improved brain cholinergic activity and neuroprotective responses, suggesting a critical role in diabetes-associated cognitive impairment [139]. Studies using both wild-type and 5XFAD mice revealed that long-term HFD feeding resulted in activation of Aβ-generating pathways and exacerbated tau pathology, particularly intensifying amyloid plaque burden in 5XFAD mice. Dietary resveratrol supplementation inhibited brain Aβ accumulation and tau pathology, significantly improving HFD-induced cognitive and behavioral impairments [140]. Furthermore, resveratrol treatment enhanced neurotrophic support by activating the SIRT1/PGC-1α/FNDC5/BDNF signaling pathway in the hippocampal CA1 region, thereby alleviating HFD-induced memory deficits [141]. Collectively, resveratrol exhibits significant efficacy in mitigating obesity-related cognitive impairment. Its multiple pathways of neuroprotective actions suggest its potential as a therapeutic candidate for preventing and managing obesity-associated cognitive decline.

Isorhamnetin (ISO) is a flavonoid extracted from the fruit of *Hippophae rhamnoides* (sea buckthorn). Recent studies have indicated that ISO mitigates cognitive dysfunction in HFFD-induced-obese mice by inhibiting MAPK and NF-κB signaling pathways [142]. Sea buckthorn flavonoids have also been shown to alleviate neuronal injury and memory deficits by modulating brain insulin signaling and inflammatory responses in mice fed a HFFDs [143]. Hydroxytyrosol, found in olive oil, has been reported to alleviate cognitive decline caused by obesity by promoting BDNF expression and support neuronal and synaptic functions by safeguarding synaptic structures in obese mice [144]. In addition, supplementation with EGCG can reverse the learning and memory impairments induced by long-term HFFD feeding, primarily through the activation of the ERK/CREB/BDNF signaling pathway [99]. PSPC also has exerted significant protective effects against obesity-induced cognitive impairment by promoting AMPK-dependent autophagic activity, reducing oxidative damage, and increasing BDNF expression, thereby attenuating neuronal apoptosis in the hippocampus [145]. Furthermore, under HFFD-induced obesity, memory function in 3xTg-AD model mice has deteriorated markedly. However, curcumin supplementation has been shown to partially reverse this damage by modulating gene enrichment patterns, particularly those involved in critical neuronal pathways such as mitochondrial organization, enhancement of excitatory postsynaptic potential, and synaptic plasticity regulation [146].

**Figure 3:**
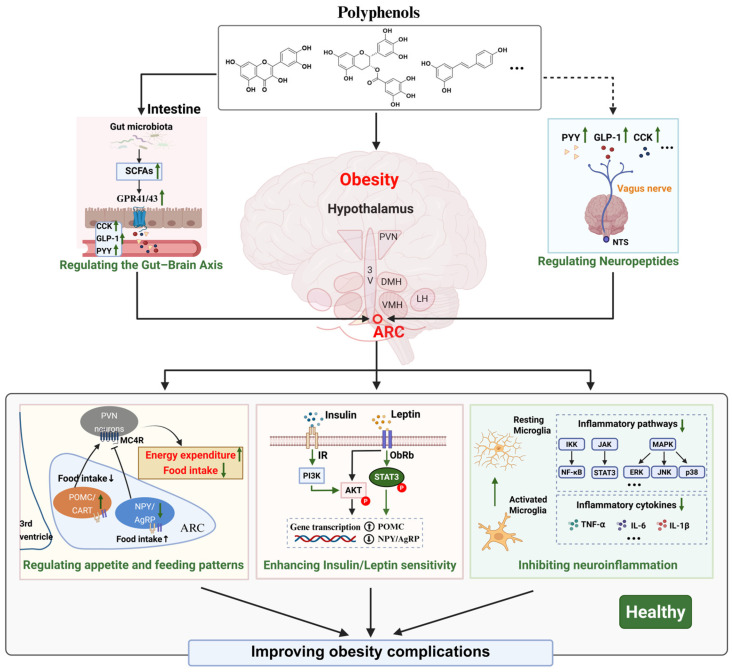
Proposed neuroregulatory mechanisms of dietary polyphenols on obesity complications. Note: In the intestine, polyphenols are metabolized by the gut microbiota to produce SCFAs, which may stimulate gut hormone secretion (e.g., GLP-1, PYY, and CCK) via GPR41/43 activation and further transmit signals to the hypothalamic arcuate nucleus (ARC) through the circulation or vagus nerve. Polyphenols may also act directly on the hypothalamus. Within the ARC, polyphenols may modulate neuropeptide expression by enhancing POMC/CART activity and suppressing NPY/AgRP signaling, thereby influencing appetite and energy expenditure. In addition, polyphenols may alleviate obesity-associated insulin/leptin resistance through the IR/PI3K/AKT and Ob-Rb/STAT3 pathways and attenuate obesity-associated neuroinflammation by inhibiting microglial activation and inflammatory pathways including NF-κB, JAK/STAT, and MAPK. Abbreviations: SCFAs, short-chain fatty acids; GPR41/43, G protein-coupled receptor 41/43; CCK, cholecystokinin; GLP-1, glucagon-like peptide-1; PYY, peptide YY; ARC, arcuate nucleus; NTS, nucleus tractus solitarius; PVN, paraventricular nucleus; DMH, dorsomedial hypothalamus; VMH, ventromedial hypothalamus; LH, lateral hypothalamus; POMC, pro-opiomelanocortin; CART, cocaine- and amphetamine-regulated transcript; NPY, neuropeptide Y; AgRP, Agouti-related peptide; MC4R, melanocortin 4 receptor; IR, insulin receptor; Ob-Rb, long form of the leptin receptor; PI3K, phosphoinositide 3-kinase. Created in https://BioRender.com.

In summary, polyphenolic compounds show considerable potential in improving obesity-related neurological disorders, primarily through appetite regulation, the gut–brain axis, preventing central neuroinflammation and brain leptin and insulin resistance, and cognitive improvement. However, bioavailability, metabolism, and BBB transport are important limiting factors in polyphenol research. Differences in bioavailability and BBB permeability among polyphenols may influence where they can enter the central nervous system and exert their effects or be retained in peripheral tissues to initiate signals to reach the brain or go through the gut–brain axis. EGCG has low bioavailability. However, it can cross the BBB and reach the brain parenchyma and exhibits its anti-obesity effects by reducing appetite. Resveratrol has extremely low bioavailability, and its neuroprotective effects may mainly depend on locally initiated intestinal signaling or its active metabolites acting in the brain to reduce inflammation and regulate leptin sensitivity. In contrast, anthocyanins and quercetin can cross the BBB and are more likely to exert direct central effects, thereby alleviating obesity-related neurological dysfunction. A summary of polyphenol subclasses, bioavailability, BBB penetration, mechanisms, and evidence levels is listed as Table 1. The proposed neuroregulatory mechanisms of dietary polyphenols on obesity complications are summarized in Figure 3.

### 3.6. Conclusions and Future Perspectives

In modern society, the excessive intake of high-carbohydrate and high-fat foods has significantly increased the prevalence of obesity, placing a heavy economic burden on global healthcare systems. Although conventional pharmacological and surgical treatments can alleviate obesity and its related complications, concerns remain regarding their long-term efficacy and potential risks. Therefore, development of safer and more effective alternative strategies is urgently needed. Dietary polyphenols, as natural health-promoting compounds, have shown promising effects in mitigating obesity complications. Recently, there has been growing interest in the neuroregulatory roles of polyphenol-rich diets on obesity complications. However, systematic and integrated analyses in this field remain limited. This review summarizes the regulatory roles of the brain in obesity complications and then reviews the effects and mechanisms by which polyphenols modulate the brain to alleviate obesity. Polyphenols may suppress HFD-induced central inflammation and neuronal damage, alleviate obesity-associated circadian rhythm disruptions, and directly regulate the expression of key appetite-related genes. Moreover, polyphenols may exert indirect effects on appetite and energy homeostasis via the gut–brain axis. However, current evidence is predominantly derived from rodent models, and there is still a lack of sufficient clinical data to validate the efficacy of polyphenols in humans, particularly long-term data. Meanwhile, the dose ranges adopted across different studies vary considerably, with certain interventions approaching pharmacological levels, while comprehensive dose–response effects need to be paid special attention. In addition, the gut–brain axis involves highly complex neural and humoral signaling networks, and the specific pathways through which polyphenols exert their effects remain largely unknown. Particularly, the downstream mechanisms involving neural circuit modulation, neurotransmitter release, and receptor activation are not yet fully understood. Chronic effects, particularly in human studies, remain underexplored. Future studies should prioritize specific polyphenols such as EGCG and resveratrol and conduct systematic investigations centered on key mechanistic endpoints, including hypothalamic microglial activation states and SCFA-mediated gut–brain signaling pathways, to provide a rational basis for the design of early-phase clinical trials. Concurrently, further investigations are required to elucidate the precise molecular mechanisms by which dietary polyphenols regulate the brain to counteract obesity. These studies should focus on identifying polyphenols’ responses hypothalamic neuronal populations, neurotransmitter systems, and receptor-mediated signaling pathways involved in appetite control and metabolic regulation while distinguishing direct central effects from secondary changes resulting from systemic metabolic improvements through brain-region-specific analyses in diet-induced obesity models. Furthermore, systematic dose-escalation studies and long-term safety evaluations are critical to define the therapeutic window of polyphenol interventions, establish minimal effective and maximal tolerated doses, and distinguish dietary intake from supplemental or pharmacological dosing. Finally, given the widespread clinical application of GLP-1 receptor agonists and dual incretin agonists in obesity management, future research should also consider potential interactions between polyphenol-based interventions and existing anti-obesity pharmacotherapies, particularly with regard to overlapping gastrointestinal effects and possible modulation of drug metabolism. It should be evaluated whether polyphenols alter the pharmacokinetic profiles of these drugs by modulating drug-metabolizing enzymes or intestinal transporters. In addition, attention should be paid to whether combined interventions may lead to additive or synergistic gastrointestinal effects, such as nausea or delayed gastric emptying. Addressing these issues will provide a stronger theoretical foundation and new directions for the development of precision obesity interventions and polyphenol-targeted therapeutic strategies. The neuron actions of polyphenols on obesity complications are summarized in Figure 3.

## Figures and Tables

**Table 1:** Summary of polyphenol subclasses, bioavailability, BBB penetration, mechanisms, and evidence Levels.

Polyphenol	Subclass	Bioavailability	BBB Penetration	Primary Mechanism	Level of Evidence	Key References
EGCG	Flavan-3-ol	Low [147]	Yes (low) [87]	Potential direct CNS	In vivo (animal)	[89,97]
				Anti-inflammatory	In vitro/in vivo (animal)	[6,99,100]
				Gut–brain axis	In vivo (animal)	[124,125,126,127]
Resveratrol	Stilbene	Very low (<1%) [148]	Negligible [149]	Anti-inflammatory	In vitro/in vivo (animal)	[103,104,105]
				Enhanced leptin sensitivity	In vivo (animal)	[118,119]
Anthocyanins	Anthocyanin	Low (2% to less than 1%) [150]	Yes (low) [149]	Anti-inflammatory	In vivo (animal)	[106,107,108,109]
				Enhanced Insulin sensitivity	In vivo (animal)	[116]
Quercetin	Flavonol	Unclear	Yes (medium) [149]	Anti-inflammatory	In vitro/in vivo (animal)	[110]

## Data Availability

Not applicable.
